# Diagnosis of treatment-related changes in children and adolescents with brain and spinal tumors: a cost-effectiveness analysis using MRI and [18 F]FET PET

**DOI:** 10.1007/s00259-025-07377-x

**Published:** 2025-06-04

**Authors:** Jurij Rosen, Jan-Michael Werner, Garry S. Ceccon, Elena K. Rosen, Michael M. Wollring, Isabelle Stetter, Philipp Lohmann, Felix M. Mottaghy, Lisbeth Marner, Ian Law, Gereon R. Fink, Karl-Josef Langen, Norbert Galldiks

**Affiliations:** 1 Department of Psychiatry, Faculty of Medicine and University Hospital Cologne, University of Cologne, Cologne, Germany; 2 Department of Neurology, Faculty of Medicine and University Hospital Cologne, University of Cologne, Kerpener St. 62, 50937 Cologne, Germany; 3 Research Center Juelich, Institute of Neuroscience and Medicine (INM-3, INM-4), Leo-Brandt-St. 5, 52425 Juelich, Germany; 4 Department of Nuclear Medicine, RWTH University Hospital Aachen, Aachen, Germany; 5 Department of Radiology and Nuclear Medicine, Maastricht University Medical Center, Maastricht, The Netherlands; 6 Center for Integrated Oncology Aachen Bonn Cologne Duesseldorf (CIO ABCD), Cologne, Germany; 7 Department of Clinical Physiology Nuclear Medicine and Nuclear Medicine, Copenhagen University Hospital Rigshospitalet, Copenhagen, Denmark; 8 Department of Clinical Physiology and Nuclear Medicine, Copenhagen University Hospital Bispebjerg, Copenhagen, Denmark

**Keywords:** Amino acid PET, Economic evaluation, Treatment monitoring, Tumor relapse

## Abstract

**Purpose:**

PET using the radiolabeled amino acid *O*-(2-[^18^F]-fluoroethyl)-L-tyrosine ([^18^F]FET) has considerable clinical value for follow-up evaluation of central nervous system tumors in children and adolescents. As medical procedures must be justified socio-economically, we determined cost-effectiveness of [^18^F]FET PET for identification of treatment-related changes.

**Methods:**

We analyzed clinical data from two different studies that assessed the value of FET PET to differentiate between brain and spinal tumor relapse and treatment-related changes in children and adolescents. Cost calculation was based on the German statutory health insurance system perspective. Due to subtle differences in the diagnostic approach of the studies, two separate clinical scenarios including 80 patients with 105 lesions were considered: Decision tree model 1 determined cost-effectiveness of simultaneous [^18^F]FET PET and MRI in comparison to MRI alone to identify treatment-related changes. Decision tree model 2 determined cost-effectiveness of [^18^F]FET PET alone to identify treatment-related changes when routine MRI findings were suspicious for tumor relapse. Deterministic and probabilistic sensitivity analyses tested the robustness of the results.

**Results:**

Model 1 revealed that the rate of identified treatment-related changes increased by 52% when adding [^18^F]FET PET to MRI, resulting in costs of €3,314.51 for each additional correctly identified lesion with treatment-related changes by [^18^F]FET PET that MRI would have misclassified. Model 2 revealed that [^18^F]FET PET correctly identified treatment-related changes in 90% of lesions when routine MRI findings were suspicious for tumor relapse, resulting in costs of €1,740.37 for each lesion.

**Conclusion:**

Integrating [^18^F]FET PET in the follow-up of in children and adolescents with brain and spinal tumor may help improving patient care at acceptable costs.

**Supplementary Information:**

The online version contains supplementary material available at 10.1007/s00259-025-07377-x.

## Introduction

Pediatric central nervous system (CNS) tumors are the most frequent solid malignancy and the most common cause of cancer death in children [1, 2]. Given the heterogeneity of tumor entities with varying malignancy and location, management of CNS tumors in this group of patients to date remains challenging. Pediatric brain gliomas constitute the majority of CNS tumors, but advances in the management of adult brain tumors cannot directly be transferred to their pediatric counterparts as these show divergent molecular features, gene expression signatures and different clinical behavior [3, 4]. Regarding diagnostic imaging, contrast-enhanced anatomical MRI is the standard imaging procedure with excellent spatial resolution and high sensitivity for tumor detection at initial diagnosis and follow-up. However, the specificity of MR imaging to reliably differentiate between neoplastic and non-neoplastic lesions is limited. In particular, the differentiation of tumor relapse from treatment-related changes, in CNS tumors using contrast-enhanced anatomical MRI alone remains challenging [5–11]. Notably, MRI signal changes such as an increase in the extent of contrast enhancement, newly occurring contrast-enhancing lesions, or an increase of signal alterations on fluid-attenuated inversion recovery sequences may reflect either treatment-related changes or tumor relapse [5–11].

Considering the limited specificity of anatomical MRI for neoplastic tissue, amino acid PET has increasingly been used to metabolically assess cerebral lesions, primarily in adults [5, 12–18], but also in children and adolescents [19–25]. Although PET using 2-[^18^F]fluoro-2-deoxy-D-glucose ([^18^F]-FDG) is the tracer of choice for numerous diagnostic approaches in patients with cancer, the Response Assessment in Pediatric Neuro-Oncology (RAPNO) Working Group has recommended the use of PET with radiolabeled amino acids such as O-(2-[^18^F]fluoroethyl)-L-tyrosine ([^18^F]FET), as it provides a higher specificity for neoplastic tissue [2].

To date, several studies have addressed the value of [^18^F]FET PET in caring for children and adolescents with CNS tumors [19–25]. Among these, Marner et al. and Dunkl et al. [19, 23] investigated the diagnostic potential of [^18^F]FET PET for differentiating between tumor relapse and treatment-related changes at follow-up. Despite subtle differences in the diagnostic approach, both studies consistently showed that [^18^F]FET PET is of considerable value for diagnosing treatment-related changes [19, 23]. Notably, the authors concluded that static and dynamic [^18^F]FET PET parameters may add valuable diagnostic information that could not be provided by anatomical MRI alone.

Nevertheless, integrating [^18^F]FET PET in the care of children and adolescents with CNS tumors is associated with additional costs that must be weighed against relevant clinical benefits for these patients, e.g., an improved diagnostic performance for the differentiation between tumor relapse and treatment-related changes. To our knowledge, no studies have yet addressed the cost-effectiveness of [^18^F]FET PET for monitoring CNS tumors in this age group. Several studies investigated the cost-effectiveness of [^18^F]FET PET imaging compared to MRI alone for increasing both the extent of resection [26] and the diagnostic yield following stereotactic biopsy [27], and for the response assessment to anticancer agents in adults with predominantly glioblastoma [28–30]. Besides, two other studies investigated the cost-effectiveness of [^18^F]FET PET for evaluating brain metastasis relapse following radiotherapy alone or multimodal therapy, including radiotherapy, targeted therapy, checkpoint inhibitors, and combinations thereof [31, 32]. These studies consistently suggested that [^18^F]FET PET use in adults is cost-effective for the clinical scenario examined. Nevertheless, these results are not directly transferable to children and adolescents as mechanisms of tumorigenesis, molecular genetic profiles, and clinical behavior of the neoplasms differ considerably between adult and pediatric patients [3, 4].

Considering the diagnostic improvements and additional costs of [^18^F]FET PET compared to anatomical MRI, the studies by Marner et al. [23] and Dunkl et al. [19] were evaluated regarding cost-effectiveness of [^18^F]FET PET to identify lesions consistent with treatment-related changes during follow-up. We performed this analysis from the perspective of the statutory health insurance system in Germany. To our knowledge, this is the first study investigating the cost-effectiveness of [^18^F]FET PET in children and adolescents suffering from CNS tumors.

## Patients and methods

### Input data

Input data were derived from the results of previously published studies by Marner et al. and Dunkl et al. that assessed the value of FET PET to differentiate between brain and spinal tumor relapse and treatment-related changes in children and adolescents [19, 23]. The institutional review board had approved these studies (Marner et al., file number: H-6–2014-095; Dunkl et al., file number: EK 022/14; clinical trial number: not applicable). Written informed consent for study participation and assessment of clinical data for scientific purposes had been obtained from the legal guardians of the minor study participants included in the study.

In the study by Marner et al., 97 children and adolescents (median age, 10 years; age range, 0.1–33 years) with 155 lesions (gliomas, 59%) were prospectively included for the evaluation of the added value of simultaneous [^18^F]FET PET and MRI compared to MRI alone [23]. In that study, inclusion criteria were suspicion or diagnosis of a primary CNS tumor before the age of 18 years, and scans were performed at initial diagnosis, before and after surgery, for response assessment, and at suspected relapse [23]. Twelve of these lesions were located at the spine. For the present analysis, only pretreated lesions were considered (i.e., radiotherapy, alkylating chemotherapy). Thus, the present analysis focuses on identifying lesions consistent with treatment-related changes in 64 patients with 83 lesions who underwent 92 FET PET and MRI scans (Supplemental Table 1).

**Fig. 1:**
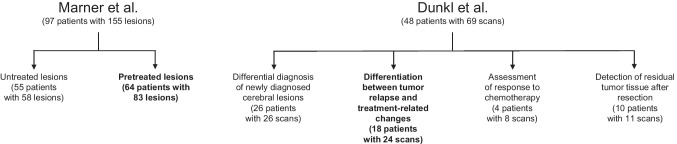
Patient selection for the cost-effectiveness analysis (in bold) from the studies of Marner et al. [23] and Dunkl et al. [19]. Note that due to exclusions and serial scanning during follow-up the numbers do not further increase

In the study by Dunkl et al., 48 children and adolescents (median age at initial diagnosis, 13 years; age range, 1–18 years) with 69 [^18^F]FET PET scans performed at different stages of the disease were retrospectively identified. All patients had been referred consecutively because decision-making for further diagnostic procedures or treatment planning was difficult using the clinical presentation or MR imaging findings alone. Specifically, FET PET scans were performed for differential diagnosis of newly diagnosed cerebral lesions, for the differentiation between tumor relapse and treatment-related changes, assessment of response to chemotherapy, or the detection of residual tumor tissue after resection (Fig. 1) [19]. For the present study, 18 patients with 24 FET PET scans that served for the differentiation between tumor relapse and treatment-related changes when routine MRI were suspicious for tumor relapse were considered (Supplemental Table 2).

### Decision tree models for the assessment of the effectiveness

Similar to earlier studies [26–30], for each clinical scenario as described by Marner et al. and Dunkl et al., one separate decision tree model was developed to assess the effectiveness, i.e. diagnostic performance, of the combined FET PET and MRI approach compared with MRI alone (Model 1: Marner et al.), and of FET PET alone (Model 2: Dunkl et al.). The calculated effectiveness subsequently served as the basis for the evaluation of cost-effectiveness, the primary outcome of the present study. Each model summarized the respective clinical scenario and neuroimaging, and assigned the examined lesions according to their respective imaging findings and, subsequently, their final diagnosis. These models were developed separately as subtle differences in the diagnostic approach of the studies warranted a distinct calculation of the effectiveness. Specifically, in model 1, FET PET and MRI were a priori conducted simultaneously, and their combined effectiveness compared to MRI alone to identify lesions consistent with treatment-related changes were compared. In model 2, a routine MRI had shown changes suspicious for tumor relapse, and prompted subsequent FET PET imaging.

Therefore, the effectiveness in model 1 was determined by comparing the effectiveness of combined FET PET and MRI with MRI alone, i.e., incremental effectiveness (IE), to identify treatment-related changes. In contrast, model 2 reflects the effectiveness of FET PET alone to differentiate tumor relapse from treatment-related changes. This different calculation method is in line with a previous study that assessed the cost-effectiveness of FET PET for the same indication in patients with brain metastases [32].

**Fig. 2:**
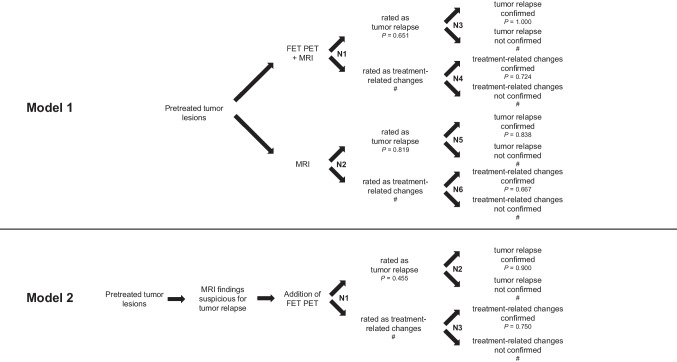
Model 1 (upper panel): Decision tree model for assessment of the effectiveness of additional [^18^F]FET PET to differentiate between tumor relapse and treatment-related changes (n=83). The model includes the two alternative strategies of using MRI alone or in combination with [^18^F]FET PET. Chance nodes N1 and N2 divide lesions in tumor relapse or treatment-related changes based on [^18^F]FET PET combined with MRI or MRI alone. Subsequent nodes N3-N6 assigned lesions with tumor relapse or treatment-related changes to their confirmed diagnosis based on neuropathological findings or the clinicoradiological follow-up. *P*-values indicate the probability that a lesion is rated as tumor relapse (N1-2) or that the respective imaging diagnosis is confirmed (N3-6). Abbreviations: #=corresponding likelihood (1-*P*); N1-6=chance nodes 1–6. Model 2 (lower panel): Decision tree for assessment of the effectiveness of additional [^18^F]FET PET to diagnose treatment-related changes (n=22) when routine MRI findings were suspicious for tumor relapse. Chance node N1 divides lesions into tumor relapse and treatment-related changes according to [.^18^F]FET PET findings. Subsequent nodes N2-3 assign lesions rated as tumor relapse or treatment-related changes to their confirmed diagnosis based on neuropathological findings or the clinicoradiological follow-up. *P*-values indicate the probability that a lesion is rated as tumor relapse (N1) or that the imaging diagnosis is confirmed (N2-3). Abbreviations: #=corresponding likelihood (1-*P*); N1-3=chance nodes 1–3

Model 1 (Fig. 2): Pretreated CNS lesions underwent both [^18^F]FET PET and MRI to differentiate between tumor relapse and treatment-related changes. Chance nodes one and two divided lesions into groups, depending on the combined [^18^F]FET PET and MRI findings (N1) or MRI findings alone (N2) (i.e., tumor relapse and treatment-related changes), respectively. The subsequent chance nodes N3-6 assigned each of these four groups of lesions rated as tumor relapse or treatment-related changes to their confirmed diagnosis.

Model 2 (Fig. 2): In pretreated CNS lesions with routine MRI findings suspicious for tumor relapse, [^18^F]FET PET was additionally performed to differentiate between tumor relapse and treatment-related changes. Chance node one (N1) divided lesions into groups depending on individual [^18^F]FET PET findings (tumor relapse and treatment-related changes). The subsequent chance nodes N2-3 assigned each of these two groups of lesions rated as tumor relapse or treatment-related changes to their confirmed diagnosis.

For both decision trees, we defined the probability of the correct identification of treatment-related changes as the primary outcome.

### Cost calculation

The costs were calculated from the perspective of the German Statutory Health Insurance system. As the German statutory health insurance companies has thus far not covered [^18^F]FET PET costs in the care of children or adolescents with CNS tumors, the costs for both [^18^F]FET PET and conventional MR imaging were calculated based on the “Medical Fee Schedule for Care Outside the Statutory Health Insurance Scheme” (http://www.e-bis.de/goae/defaultFrame.htm) to provide an equal and consistent determination of the cost.

As described previously [29], the costs taken into consideration for [^18^F]FET PET were as follows (procedure’s index number in parenthesis): Patient consultation €10.72 (1), report on diagnostic findings €17.43 (75), intravenous injection €9.38 (253), scintigraphy of the brain €125.91 (5430), [^18^F]FET PET with quantitative analysis €786.89 (5489), and tracer production costs of €616.00. For MRI, the expenses were as follows: Patient consultation €10.72 (1), physical examination €10.72 (5), report on diagnostic findings €17.43 (75), high-pressure intravenous injection €40.23 (346), surcharge for perfusion imaging €75.19 (3051), MRI with three-dimensional and apparent diffusion coefficient (ADC) reconstruction requiring substantial technical effort €641.16 (5700), additional MRI series with three-dimensional and ADC reconstruction requiring substantial technical effort €145.72 (5731), and surcharge for computer analysis €46.63 (5733). Thus, the imaging costs for one [^18^F]FET PET were estimated at €1,566.33 and €987.80 for one MRI scan.

In decision tree model 1, the identification of a lesion consistent with treatment-related changes comprised one [^18^F]FET PET and one MRI scan. Thus, the costs of both imaging modalities were added for cost calculation. Moreover, as 92 scans were performed for 83 treated lesions, an additional factor (i.e., 92/83) for the cost calculation was considered, resulting in imaging costs of €2,831.08 per lesion. In decision tree model 2, the identification of a lesion consistent with treatment-related changes comprised one [^18^F]FET PET scan. Therefore, the imaging costs per lesion resulted in €1,566.33.

### Cost-effectiveness

In decision tree model 1, the effectiveness of the correct identification of treatment-related changes was compared between the combined [^18^F]FET PET and MRI approach, and MRI alone (i.e., incremental effectiveness (IE)). Hence, the difference in cost between [^18^F]FET PET combined with MRI, and MRI alone divided by the IE resulted in the incremental cost-effectiveness ratio (ICER):

$$\begin{array}{l}\mathrm{ICER}=\\\begin{array}{lc}\frac{\mathrm{Cost}\left(\left[18\mathrm F\right]\mathrm{FET}\;\mathrm{PET}+\mathrm{MRI}\;\right)-\mathrm{Cost}\;\left(\mathrm{MRI}\right)}{\mathrm{Effectiveness}\left(\left[18\mathrm F\right]\mathrm{FET}\;\mathrm{PET}+\mathrm{MRI}\;\right)-\mathrm{Effectiveness}\;\left(\mathrm{MRI}\right)}\end{array}\end{array}$$

In decision tree model 2, the effectiveness of correct identification of treatment-related changes was calculated for [^18^F]FET PET alone. Thus, the cost for one FET PET divided by its effectiveness resulted in the cost-effectiveness ratio (CER):

$$\text{CER}=\frac{\text{Cost }\left([18\text{F}]\text{FET PET}\right)}{\text{Effectiveness }([18\text{F}]\text{FET PET})}$$

### Sensitivity analyses

Deterministic and probabilistic sensitivity analyses were performed to test the robustness of the calculated effectiveness. In particular, one-way deterministic sensitivity analysis evaluated the impact of each independent variable (model 1, chance nodes N1-6; model 2, chance nodes N1-3) on the resulting effectiveness and, thus, the (incremental) cost-effectiveness ratio. Due to a lack of previous studies evaluating the cost-effectiveness of [^18^F]FET PET in children and adolescents with CNS tumors, and since the majority of analyzed lesions were gliomas, available confidence intervals already used in comparable studies that evaluated the cost-effectiveness of [^18^F]FET PET for treatment monitoring in adult patients with gliomas were applied to each variable (Table 1) [28–30]. In model 1, as the calculated value for N3 was 100%, changing that chance node value within the deterministic sensitivity analysis resulted in theoretical values > 100%. Thus, the resulting IE and ICER based on these values are likewise considered theoretical.

**Table 1:** Chance node intervals and corresponding effectiveness and CER in the one-way deterministic sensitivity analysis for decision tree models 1 and 2

Chance node	Parameter	Decision tree model 1		Decision tree model 2	
		lower interval	upper interval	lower interval	upper interval
	Value (%)	50	80	31	61
N1	Effectiveness (%)	52	52	95	83
	CER (€)	3,314.51	3,314.51	1,657.78	1,885.60
	Value (%)	67	97	83	98
N2	Effectiveness (%)	33	88	84	97
	CER (€)	5,271.88	1,962.97	1,870.89	1,609.84
	Value (%)	93	108*	68	83
N3	Effectiveness (%)	36	76*	89	91
	CER (€)	4,794.30	2,276.21*	1,759.70	1,724.55
	Value (%)	65	80		
N4	Effectiveness (%)	52	52	n.a	n.a
	CER (€)	3,314.51	3,314.51		
	Value (%)	76	91		
N5	Effectiveness (%)	62	37	n.a	n.a
	CER (€)	2,814.54	4,678.84		
	Value (%)	59	74		
N6	Effectiveness (%)	55	50	n.a	n.a
	CER (€)	3,136.95	3,492.08		

The effectiveness and the cost-effectiveness ratio were calculated based on the indicated change node values. Note that (i) values for the effectiveness and the cost-effectiveness ratio of decision tree model 1 correspond to incremental values (difference between [^18^F]FET PET+MRI and MRI alone), and (ii) decision tree model 2 comprises only chance nodes N1-3. Abbreviations: *CER* cost-effectiveness ratio; *n.a.* not applicable

^*^theoretical values as N3>100%

For probabilistic sensitivity analysis, a Monte Carlo analysis was performed using 10,000 sets of positive random values for the independent variables (Model 1, chance nodes N1-6; Model 2, chance nodes N1-3). The distribution of these random values was defined by the mean of the decision trees and the standard deviation, which was set according to the respective confidence interval of the deterministic sensitivity analysis, similar to earlier studies [28, 29] (Table 2). As described previously [29], for each set of random values, we determined the effectiveness separately for both [^18^F]FET PET and MRI, and their respective difference (i.e., IE) (Model 1), or the effectiveness of [^18^F]FET PET alone (Model 2). The (incremental) cost-effectiveness ratio was determined based on the effectiveness values.

**Table 2:** Values for decision tree models 1 and 2 and Monte Carlo analysis

Chance node	Decision tree model 1		Decision tree model 2	
	Value (%)	SD (%)	Value (%)	SD (%)
N1	65	8	46	8
N2	82	8	90	4
N3	100	4	75	4
N4	72	4	n.a	n.a
N5	84	4	n.a	n.a
N6	67	4	n.a	n.a

The values for the chance nodes were calculated based on the data by Marner et al. (Model 1) and Dunkl et al. (Model 2). Standard deviations were set according to the confidence intervals of the deterministic sensitivity analysis, as reported previously [28, 29]. Note that decision tree model 2 comprises only chance nodes N1-3. Abbreviations: *SD* standard deviation; *n.a.* not applicable

Furthermore, imaging costs were modeled by a gamma distribution with the mean imaging cost per lesion and a standard deviation of 50% of the corresponding mean. The probabilistic sensitivity analysis results for effectiveness values were displayed by mean, median, standard deviation, 95% confidence interval (CI), minimum and maximum values, and the 2.5 th-, 10 th-, 90 th-, 97.5 th-percentiles. All calculations, Figures, and simulations were performed using the statistical computing language and environment software R [33–35].

## Results

### Effectiveness

Decision tree model 1 revealed that adding [^18^F]FET PET to MRI increased the fraction of correctly identified treatment-related changes by 52% (correct identification by [^18^F]FET PET and MRI vs. MRI alone, 100% vs. 48%). Thus, two lesions had to be examined to identify one additional lesion consistent with treatment-related changes using [^18^F]FET PET. Decision tree model 2 revealed that [^18^F]FET PET correctly identified treatment-related changes in 90% of lesions when routine MRI findings were suspicious for tumor relapse. Thus, two lesions had to be examined to identify one lesion consistent with treatment-related changes by [^18^F]FET PET. Calculated values for the chance nodes of both decision trees are indicated in Fig. 2 and Table 2.

### Cost-effectiveness

For decision tree model 1, the ICER, i.e., the cost to correctly identify one additional lesion consistent with treatment-related changes by adding [^18^F]FET PET to MRI that would have been misclassified using MRI alone, resulted in €3,314.51. For decision tree model 2, the CER, i.e., the cost of correctly identifying one additional lesion consistent with treatment-related changes when routine MRI findings were suspicious for tumor relapse, resulted in €1,740.37.

### Sensitivity analyses

**Fig. 3:**
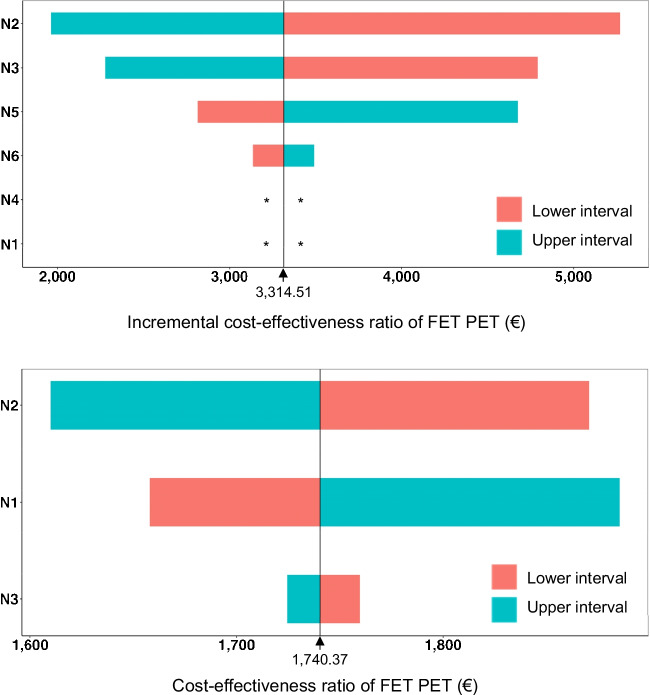
Tornado diagrams of the (incremental) cost-effectiveness ratio of [^18^F]FET PET for the identification of treatment-related changes (Model 1, upper panel), and treatment-related changes when routine MRI findings were suspicious for tumor relapse (Model 2, lower panel). The indicated (incremental) cost-effectiveness ratios resulted from applying the upper and lower interval values of the one-way deterministic sensitivity analysis onto change nodes N1-6 and N1-3, respectively. *Applying interval values on N4 and N1 did not affect the resulting incremental cost-effectiveness ratios

**Fig. 4:**
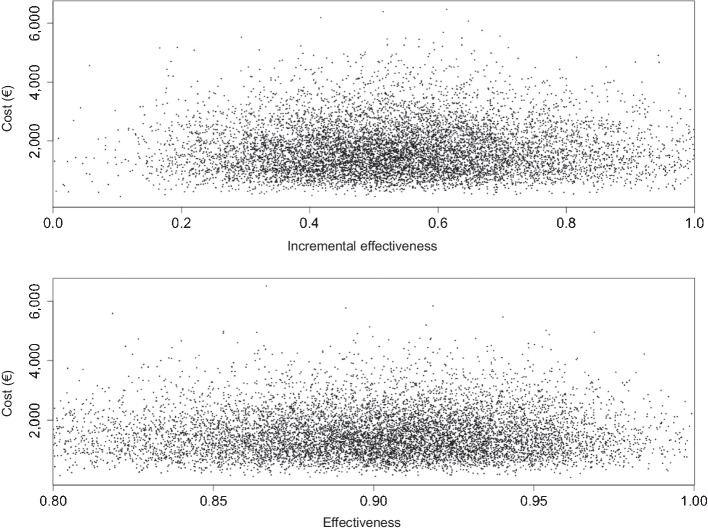
Distribution of results from Monte Carlo analysis (dots) about the effectiveness of [^18^F]FET PET for the identification of treatment-related changes (Model 1, upper panel) and treatment-related changes when routine MRI findings were suspicious for tumor relapse (Model 2, lower panel). Note that the x-axes of the panels are differently scaled. Margin values are not shown

For decision tree model 1, the resulting IE and ICER for the chance node intervals of the deterministic sensitivity analysis are presented in Table 1. The upper panel in Fig. 3 shows the corresponding Tornado diagram. The range of ICER values was €1,962.97—€5,271.88. The results for both the IE and ICER of the probabilistic sensitivity analysis exceeded the calculated values of the decision tree (mean IE, 55%; 95% CI, 58—68%; mean ICER, €3,146.05; 95% CI, €2,545.75—€3,009.63) (Table 3; upper panel in Fig. 4).

**Table 3:** Statistics resulting from the Monte Carlo analysis, including 10,000 samples for the effectiveness

Value/ Percentile	Decision tree model 1				Decision tree model 2	
	MRI (%)	[^18^F]FET PET + MRI (%)	IE (%)	Cost for additional [^18^F]FET PET (€)	[^18^F]FET PET (%)	Cost for additional [^18^F]FET PET (€)
Mean	46	101*	55*	1,719.62	90	1,551.39
SD	15	13		862.13	5	777.79
Minimum	−91*	59	150*	98.65	66	89.00
2.5 th	13	81	68	488,74	80	440.92
10 th	27	89	62	759.79	84	685.46
Median	48	100	52	1,573.98	90	1,420.00
90 th	64	115*	51*	2,865.63	95	2,585.29
97.5 th	71	129*	58*	3,814.70	97	3,441.53
Maximum	96	430*	335*	7,222.32	104*	6,515.79

Columns indicate the probability of correct identification of treatment-related changes (Model 1), and treatment-related changes when routine MRI findings were suspicious for tumor relapse (Model 2) by the indicated imaging modality. Column IE indicates the difference of probabilities and thus the incremental effectiveness in using [^18^F]FET PET. Column Cost for additional [^18^F]FET PET indicates the gamma-distributed additional [^18^F]FET PET imaging cost per lesion. Abbreviations: *IE* incremental effectiveness; *SD* standard deviation

^*^ theoretical values

For decision tree model 2, the resulting effectiveness and CER for the chance node intervals of the deterministic sensitivity analysis are presented in Table 1. The lower panel in Fig. 3 shows the corresponding Tornado diagram. The range of CER values was €1,609.84—€1,885.60. The results of the probabilistic sensitivity analysis showed both a narrow distribution around the mean and a close relation to the calculated effectiveness and CER values of the decision tree (mean effectiveness, 90%; 95% CI, 80–97%; mean CER, €1,743.94; 95% CI, €1,608.53—€1,968.27) (Table 3; lower panel in Fig. 4). This close relation confirmed the robustness and reliability of the calculated values of the decision tree.

## Discussion

The main finding of the present study is that [^18^F]FET PET was cost-effective in identifying treatment-related changes in children and adolescents with pretreated CNS tumors.

The robustness of the results was supported by the comparatively small range of the deterministic and probabilistic sensitivity analysis results. Besides, for decision tree 1, the results of the probabilistic sensitivity analysis suggested even higher values for the IE, with a more favorable mean of 55% and a 95% CI of 58–68%, which exceeded the calculated value of the decision tree (i.e., 52%). The fact that the calculated values were outside the indicated CI may raise questions regarding the robustness of the results. Nevertheless, this deviation was merely a result of the calculation method of probabilistic sensitivity analysis. In any case, these results reflected the diagnostic value of [^18^F]FET PET for identifying treatment-related changes in this patient group.

The clinical scenarios illustrated in the decision tree models 1 and 2 showed differences in the analyzed patient groups regarding tumor types, pretreatment, and the temporal sequence of neuroimaging. In both scenarios, our results were based on identifying treatment-related changes since this identification considerably influences further treatment planning in affected patients. This particularly applied to clinical situations in which treatment-related changes such as pseudoprogression on MRI may lead to a discontinuation of a potentially effective treatment based on the false assumption of tumor relapse. Thus, a premature and more aggressive treatment regimen, including surgical interventions, with the risk of severe side effects and a decrease in health-related quality of life, can be avoided.

From an economic point of view, the exact amount of potentially saved cost due to an avoidance of a probably more costly and more aggressive treatment option by adding [^18^F]FET PET to the diagnostic work-up was almost impossible to assess given the number of available treatment options for various CNS tumor types in the evaluated groups of pediatric and adolescent patients. For glioblastomas, the overall cost of standard treatment consisting of radiotherapy with concomitant and six cycles of adjuvant temozolomide chemotherapy is approximately €30,000 [36, 37], the comparatively lower expense for [^18^F]FET PET for differentiating between tumor relapse and treatment-related changes seems to be cost-effective.

In comparison to other studies evaluating the cost-effectiveness of [^18^F]FET PET for assessment of tumor lesions in adult patients with gliomas compared to MRI, the calculated ICER of decision tree 1 was lower and thus more favorable, confirming the cost-effectiveness of [^18^F]FET PET for the identification of treatment-related changes in this patient group [27, 29, 30]. Similarly, compared to two other studies that investigated the cost-effectiveness of [^18^F]FET PET for evaluating brain metastasis relapse following radiotherapy alone or multimodal therapy, including radiotherapy, targeted therapy, checkpoint inhibitors, and combinations thereof, the calculated ICER of decision tree 1 was lower [31, 32]. Only one study that investigated the cost-effectiveness of [^18^F]FET PET for assessing treatment response in patients with gliomas showed lower results for the ICER [28]. This difference was likely due to differences in the cost calculation that was done in the context of Belgian healthcare system in the mentioned study, while the underlying results for the IE were similar to the present results [28]. In principle, comparability of the results was limited as the studies above exclusively investigated the cost-effectiveness of [^18^F]FET PET in the care of adult patients, and the assessed clinical scenarios differed from the scenarios assessed in the present study.

Other advanced imaging modalities, such as diffusion- or perfusion-weighted MR imaging, and proton magnetic resonance spectroscopy can similarly contribute further details on the underlying biology of tumors, particularly regarding molecular, physiological, and functional characteristics [38]. With regard to perfusion MRI, its diagnostic accuracy appears to be inferior compared to FET PET for differentiating tumor relapse and treatment-related changes in adult patients [39]. Moreover, advanced MR imaging remains relatively non-standardized, as outputs are considerably influenced by scanner-specific parameters, particularly the choice and configuration of sequences [40]. In contrast, FET PET acquisition has been standardized [41], allowing for more consistent and comparable results across different institutions. Furthermore, the value of advanced MRI techniques was predominantly assessed in adult patients, and data obtained in pediatric patients with brain tumors remain still scarce.

There are a number of limitations of the present study that warrant further consideration. First, although the majority of the assessed tumor lesions were gliomas, the tumors evaluated in our study are to a certain degree heterogenous in terms of neuropathological diagnosis and location, such as spinal lesions. Nevertheless, we did not exclude these lesions from the analysis since diagnostic and therapeutic interventions are widely comparable. In particular, the value of additional FET PET for identifying treatment-related changes has been demonstrated for all types of lesions in the studies on which our analysis is based. Secondary brain tumors were not included. Second, a putative limitation of the present results for the cost-effectiveness ratios was their focus on individual lesions. This aspect needs to be considered in patients with multiple lesions as these result in repetitive scans and consequently higher expenses. Notwithstanding, by focusing on the individual lesion, the present results are more generalizable to other patients as the number of lesions naturalistically varies between patients. In addition, we considered the number of scans for each individual lesion to realistically reflect increased cost for serial scanning. The third limitation was that the cost calculation was conducted within the context of a specific, i.e., German healthcare system, using a similar approach to evaluate (cost-) effectiveness of [^18^F]FET PET for other indications in patients with brain tumors as described earlier [26–31]. Consequently, the present results for the (incremental) cost-effectiveness ratios could not directly be transfered to other countries due to national differences in healthcare and cost structures. For example, in Belgium, cost for [^18^F]FET PET scanning are considerably lower (cost for one [^18^F]FET PET approximately €400) due to national grants, distinct reimbursement schemes, and dependency of cost from the number of annually performed [^18^F]FET PET scans [28]. Of note, if the Belgian scanning cost were taken as a basis for the present analysis, the resulting cost-effectiveness of [^18^F]FET PET would probably have been even more favorable [28]. In addition, the present results for the (incremental) effectiveness (i.e., the probability of correctly identifying treatment-related changes) appeared to be transferable to other countries as these predominantly depended on the information obtained from the respective neuroimaging modality. Thus, the present data showing an (incremental) effectiveness may help physicians choose the most appropriate neuroimaging approach during follow-up and considerably facilitate further cost evaluations from the perspective of health economics.

## Conclusion

This study suggests that [^18^F]FET PET is cost-effective for identification of treatment-related changes in pretreated CNS tumors in children and adolescents and improves patient care at acceptable costs.

## Supplementary Information

Below is the link to the electronic supplementary material.

Supplementary file1 (DOCX 35 KB)

## Data Availability

The datasets generated during and/or analyzed during the current study are available from the corresponding author on reasonable request.
