# Supplementary material for: Diagnosis of treatment-related changes in children and adolescents with brain and spinal tumors: a cost-effectiveness analysis using MRI and [18 F]FET PET
Source: Eur J Nucl Med Mol Imaging. 2025 Jun 4;52(12):4616–26. doi: 10.1007/s00259-025-07377-x (PMC12491346; doi:10.1007/s00259-025-07377-x)
Supplement: Supplementary file 1 — Supplementary file1 (DOCX 35 KB) [file 259_2025_7377_MOESM1_ESM.docx]

**Supplemental Table 1:** Patient demographics, distribution of diagnoses, and pretreatment in the study of Marner et al. [1]

| **Demographics** |  |  |
| --- | --- | --- |
| Patients (n); lesions (n) | 64; 83 |  |
| Sex (female) | 47% |  |
| Age at FET PET imaging (median; range) | 9 years; 0-33 years |  |
|  |  |  |
| **Diagnoses** | **Lesions (n)** | **WHO grades** |
| Anaplastic glioma | 1 | III |
| Anaplastic pilocytic astrocytoma | 2 | III |
| Anaplastic pleomorphic xanthoastrocytoma | 1 | III |
| Astrocytoma | 1 | II |
| Atypical plexus papilloma | 1 | II |
| Atypical teratoid rhabdoid tumor | 4 | IV |
| Craniopharyngioma | 1 | n.a. |
| Diffuse midline glioma, H3K27M mutant | 8 | IV |
| Dysembryoplastic neuroepithelial tumor | 1 | I |
| Ependymoma | 10 | II, III |
| Ganglioglioma | 6 | I |
| Glioblastoma | 3 | IV |
| High-grade neuroepithelial tumor with MN1 alteration | 1 | I |
| Juvenile xanthogranuloma | 1 | n.a. |
| Malignant germ cell tumor | 1 | IV |
| Medulloblastoma (non-WNT/non-SHH) | 1 | IV |
| Neurocytoma | 1 | II |
| Oligodendroglioma | 1 | II |
| Pilocytic astrocytoma | 31 | I |
| Pilocytic astrocytoma/Ganglioglioma* | 3 | I |
| Pineoblastoma | 1 | IV |
| Pleomorphic xanthoastrocytoma | 2 | II |
| Teratoma | 1 | IV |
|  |  |  |
| **Pretreatment** | **Lesions (n)** |  |
| Surgery only | 42 |  |
| Radiation therapy | 4 |  |
| Chemotherapy | 25 |  |
| Radiation and chemotherapy | 12 |  |

**Abbreviations:** **n** = number; **n.a.** = WHO grade not assessed; **SHH** = Sonic Hedgehog; **WNT** = Wingless; * = tumor described as a combination with features of both tumor types

**Supplemental Table 2:** Patient demographics, distribution of diagnoses, and pretreatment in the study of Dunkl et al. [2]

| **Demographics** |  |  |
| --- | --- | --- |
| Patients (n); lesions (n) | 16; 22 |  |
| Sex (female) | 63% |  |
| Age at FET PET imaging (median; range) | 12 years, 0-17 years |  |
|  |  |  |
| **Diagnoses** | **Lesions (n)** | **WHO grades** |
| Anaplastic astrocytoma | 1 | III |
| Astrocytoma | 5 | I, II |
| Ependymoma | 2 | II, III |
| Glioblastoma | 8 | IV |
| Medulloblastoma | 1 | IV |
| Oligoastrocytoma | 2 | III |
| Oligodendroglioma | 2 | II |
| Pleomorphic xanthoastrocytoma | 1 | II |
|  |  |  |
| **Pretreatment** | **Lesions (n)** |  |
| Surgery only | 6 |  |
| Chemotherapy | 3 |  |
| Radiation and chemotherapy | 11 |  |
| Proton therapy | 2 |  |

**Abbreviations: n** = number

**REFERENCES**

1. Marner L, Lundemann M, Sehested A, Nysom K, Borgwardt L, Mathiasen R, et al. Diagnostic accuracy and clinical impact of [18F]FET PET in childhood CNS tumors. Neuro Oncol. 2021;23:2107-16. doi:10.1093/neuonc/noab096.

2. Dunkl V, Cleff C, Stoffels G, Judov N, Sarikaya-Seiwert S, Law I, et al. The usefulness of dynamic O-(2-18F-fluoroethyl)-L-tyrosine PET in the clinical evaluation of brain tumors in children and adolescents. J Nucl Med. 2015;56:88-92. doi:10.2967/jnumed.114.148734.
